# Diagnosis of Canine Leptospirosis by a Highly Sensitive FRET-PCR Targeting the *lig* Genes

**DOI:** 10.1371/journal.pone.0089507

**Published:** 2014-02-24

**Authors:** Chuanling Xu, Amanda Loftis, Sudhir K. Ahluwalia, Dongya Gao, Ashutosh Verma, Chengming Wang, Bernhard Kaltenboeck

**Affiliations:** 1 Department of Pathobiology, College of Veterinary Medicine, Auburn University, Auburn, Alabama, United States of America; 2 Department of Biomedical Sciences, Ross University School of Veterinary Medicine, St. Kitts, West Indies; Federal University of Pelotas, Brazil

## Abstract

Canine leptospirosis is underdiagnosed due to its wide spectrum of clinical presentations and the lack of a rapid and sensitive test for the accurate diagnosis of acute and chronic infections. In this study, we developed a highly sensitive and specific fluorescence resonance energy transfer (FRET)-PCR to detect common pathogenic leptospires in dogs, including *Leptospira interrogans* serovars Autumnalis, Canicola, Copenhageni (Icterohaemorrhagiae serogroup) and Pomona, and *Leptospira kirschneri* serovar Grippotyphosa. This PCR targets the *lig* genes, exclusively found in the pathogenic *Leptospira* species but not in saprophytic species (*L. biflexa*). A robust, high-stringency step-down real-time platform was coupled to the highly specific detection of leptospiral DNA by fluorescently labeled FRET probes. This enabled the detection of a single copy of the *lig* gene in a PCR containing DNA from up to 50 µL canine blood or 400 µL urine. Sensitivity determination by use of limiting serial dilutions of extracted leptospiral DNA indicated that the *lig* FRET-PCR we established was almost 100-fold more sensitive than the widely accepted lipL32 SYBR assay and 10-fold more sensitive than a 16S rRNA TaqMan assay. Application of this method to 207 dogs with potential leptospiral infection enabled us to diagnose three cases of canine leptospirosis characterized by low amounts of leptospiral DNA in body fluids. Detection of canine leptospirosis with the *lig* FRET-PCR was more sensitive with the *lig* FRET-PCR than with the 16S rRNA TaqMan PCR, which detected only 2 of the 3 cases, and the lipL32 SYBR PCR, which detected none of the 3 dogs with leptospirosis.

## Introduction

Leptospirosis is a zoonotic disease caused by pathogenic leptospires that infect a wide range of animals around the world [1]–[3]. The clinical diagnosis of canine leptospirosis is difficult because multiple factors, including the virulence of the infecting leptospires, environmental factors affecting the organisms, and the age and immune status of the dog, result in a broad spectrum of clinical manifestations and laboratory findings [1]–[3]. Antibody-based assays can be used for diagnosis, but they have limited sensitivity in the early stage of illness, and antibodies elicited by vaccination cannot be discriminated from natural infection. Since 1986, molecular techniques such as southern blotting, dot-blotting [4], *in situ* hybridization [5], and nucleic acid amplification techniques such as real-time PCR assays [6]–[10] have been applied to detect the presence of leptospiral nucleic acids. Quantitative real-time PCR assays targeting the genes *secY*, *lfb1*, *lipL32*, *lig*, and using either SYBR green or hydrolysis probes (such as TaqMan) for detection, have been widely used for the diagnosis of leptospirosis [6]–[13].

Before 1989, the genus *Leptospira* was divided into two species, *L*. *interrogans*, including all pathogenic leptospires, and *L. biflexa,* including all saprophytic leptospires. Currently, based on genetic relatedness, the genus *Leptospira* has been reclassified into 22 species (11 pathogenic), about 38 antigenically related serogroups (31 pathogenic) and more than 250 serovars [1]–[3]. Unfortunately, the leptospiral genomes are highly fluid and contain a high number of transposases to mediate multiple genome rearrangements [2], which makes it challenging to design highly sensitive and specific real-time PCR assays to accurately quantitate and differentiate all pathogenic and saprophytic leptospiral strains. The most common pathogenic leptospires in dogs are *L. interrogans* serovars Canicola, Icterohemorrhagiae, and Pomona, and *L. kirschneri* Grippotyphosa [1]–[3], [14]. Serovars Canicola and Icterohaemorrhagiae are the primary pathogenic strains associated with canine leptospiral disease [1].

The leptospiral immunoglobulin-like (*lig*) gene produces Lig proteins that are key virulence determinants in pathogenic *Leptospira* species [15]. These *lig* genes are only present in pathogenic, but not saprophytic, *Leptospira* species [10], [15]. Three *lig* genes, designated *ligA*, *ligB*, and *ligC* (pseudogene in many species), have been identified. The amino terminal domains of the LigA and LigB paralogs are essentially identical, showing 98.5% similarity. In this report, we describe the development and application of a highly sensitive FRET-PCR targeting a region of the *lig* gene which is identical for *ligA* and *ligB* genes.

## Materials and Methods

### Dogs

All work in this study was approved by the Institutional Animal Care and Use Committee of Ross University School of Veterinary Medicine (RUSVM). Written or verbal consent from the owners of the dogs was received to use blood and urine in this study. Whole blood collected in ethylenediaminetetraacetic acid (EDTA) was used for complete blood count (CBC) and comprehensive biochemical profiles using the VetScan HM5 and VetScan VS2 platforms (Abaxis, Union City, CA). Urine samples were tested using a VetLab UA Analyzer (IDEXX, Westbrook, ME) and proteinuria was further confirmed by the use of sulfosalicylic acid (SSA) precipitation. For PCR testing, urine (up to 4 mL) was centrifuged for 5 minutes at 10,000 g and the resulting pellet was resuspended in 200 µL of phosphate-buffered saline. Aliquots of 200 µL of whole blood or 200 µL of urine sediment were frozen at −20°C until DNA extraction was performed.

### DNA Extraction

The High-Pure PCR Template Preparation Kit (Roche Molecular Biochemicals, Indianapolis, IN) was used to extract total nucleic acids from blood and urine samples as previously described [16]. The blood or urine sample was mixed with an equal volume of binding buffer [6 M guanidine-HCl, 10 mM urea, 20% (v/v) Triton X-100, 10 mM Tris-HCl, pH 4.4], and the DNA samples were eluted in 40 µL of elution buffer for blood and 100 µL for urine.

### Real-time PCRs

The widely used lipL32 SYBR and 16S rRNA TaqMan assays to detect leptospiral DNA were performed on an Applied Biosystems 7500 Real-time PCR System (Applied Biosystems, Foster City, CA, USA) as published [7], [9]. FRET-PCR assays for *Anaplasma* 16S rRNA and *Babesia* 18S rRNA were performed on a Roche LightCycler 2.0 PCR Instrument (Roche Applied Science, Indianapolis, IN, USA) as previously described [17]. The hydrolysis-probe based PCR for *Ehrlichia canis* 16S rRNA was performed on an Applied Biosystems 7500 Real-time PCR System (Applied Biosystem, Foster City, CA) as described previously [18].

**Figure 1:**
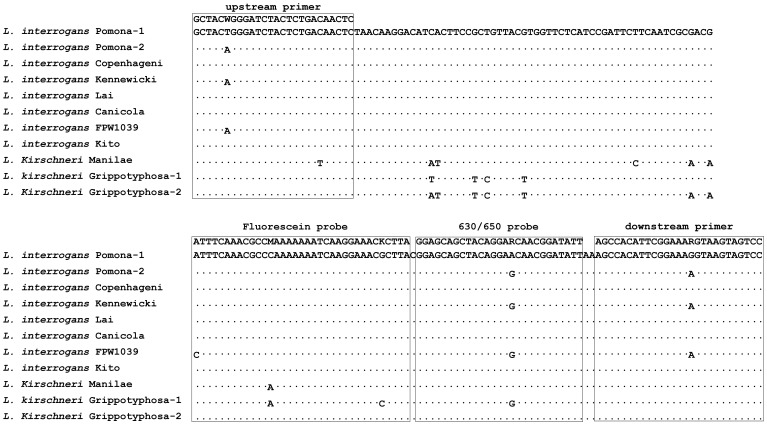
Alignment of the FRET-PCR amplicons from the partial *lig* gene. Dots indicate nucleotides identical to the *L. interrogans* serovar Pomona-1 *lig* gene (gene accession number AF534640.1). Primers and probes are indicated by boxes. The upstream primer and two probes are used as the indicated sequences without gaps while the downstream primer is used as an antisense oligonucleotide.

In this study, we designed a FRET-PCR to target the leptospiral *lig* genes [15]. The nucleotide sequences of the *lig* genes of the pathogenic leptospires for dogs were obtained from GenBank. The primers and probes for the *lig* FRET-PCR were designed by use of Vector NTI software (Invitrogen Corporation, Carlsbad, CA) and synthesized by Integrated DNA Technologies (Coralville, IA). The *lig* FRET-PCR amplifies a 176-bp target, and the positions of primers and probes are shown in Fig. 1. The forward and reverse primers and the anchor and reporter probes have 1, 2, 1, and 1 degenerate nucleotides, respectively: forward primer: 5′-GCTACWGGGATCTACTCTGACAACTC-3′; reverse primer: 5′-GGACTACTTACYTTTCCGAATGTGGCT-3′; anchor probe: 5′- ATTTCAAACGCCMAAAAAAATCAAGGAAACKCTTA-(6-FAM)-3′; reporter probe: 5′-(Bodipy630/650)-GGAGCAGCTACAGGARCAACGGATATT-(Phosphate)-3′. The fluorescein probe was 3′-labeled with carboxyfluorescein (6-FAM) and acts as the FRET donor probe, excited by 488 nm light. The Bodipy630/650 probe was HPLC-purified and is used as the FRET acceptor probe, emitting ∼640 nm fluorescence following excitation by 6-FAM in close physical proximity. To ensure the specificity of the *lig* FRET-PCR, all four oligonucleotides were analyzed for similarity with other sequences by nucleotide megablast (optimized for highly similar sequences) versus all accessioned sequences in GenBank.

FRET-PCR was performed on a LightCycler 2.0 real-time PCR platform (Roche Molecular Biochemicals, Indianapolis, IN) using a previously described thermal protocol and PCR conditions [16]. Each reaction was performed in a 20 µL final volume, using 10 µL of extracted DNA. Thermal cycling consisted of 18 high-stringency step-down cycles followed by 25 relaxed-stringency fluorescence acquisition cycles. The 18 high-stringency step-down thermal cycles were 6×0 sec @ 95°C, 12 sec @ 64°C, 8 sec @ 72°C; 9×0 sec @ 95°C, 12 sec @ 62°C, 8 sec @ 72°C; 3×0 sec @ 95°C, 12 sec @ 60°C, 8 sec @ 72°C. The relaxed-stringency fluorescence acquisition cycling consisted of 30×0 sec @ 95°C, followed by fluorescence acquisition of 12 sec @ 58°C, and 10 sec @ 72°C. Following the completion of FRET-PCR, the melting curve analysis for probes annealing to the PCR products was determined by monitoring the fluorescence from 37°C to 85°C, and the first derivatives of F4/F1 were evaluated to determine the probe melting temperature (*T*_m_) [16].

DNA from a pure culture of a wild-type isolate of *Leptospira interrogans* serovar Pomona (a gift from Frank Austin of Mississippi State University to Auburn University) was extracted and quantified by PicoGreen DNA fluorescence assay (Invitrogen Corporation, Carlsbad, CA). The extracted and quantitated DNA was used as a positive control and quantitative standard for the *lig* FRET-PCR. Numbers of genomes were calculated assuming a *Leptospira* spp. genome size of 4.63 Mb (MW = 3.06×10^9^ Dalton). For preparation of quantitative standards, the *Leptospira* genomic DNA was diluted in a background of 20 µg/mL salmon sperm DNA in TE buffer. This genomic DNA standard was used to determine the sensitivity of the *lig* FRET-PCR by amplification of logarithmic dilutions of the standard. To calculate the actual number of *lig* gene targets per leptospiral genome by limiting dilution analysis, the 10-copy standard DNA was further serially diluted from 1∶100-fold (0.1 genomes/PCR) to 1∶500-fold (0.02 genomes/PCR), and 5 aliquots of each dilution step were subjected to PCR detection. As negative control, the standard dilution buffer containing 20 µg/mL salmon sperm DNA was used supplemented with unrelated bacterial DNAs from *E. coli*, *Pseudomonas aeruginosa*, and *Bordetella bronchiseptica*. For specificity assurance, the products of *lig* FRET-PCR were verified by Sanger sequencing of both strands (Davis Sequencing, Davis, CA, USA).

To compare the sensitivity and specificity of the *lig* FRET-PCR with the published SYBR Green PCR for detection of the *lipL*32 gene and the TaqMan PCR for detection of the 16S rRNA gene, DNA from pure cultures of five different strains of pathogenic *Leptospira interrogans* (serovar Autumnalis, strain Akiyami A; serovar Canicola, strain Hond Utrecht IV; serovar Copenhageni (ictero reference), strain M-20; serovar Pomona, strain Pomona) and *Leptospira kirschneri* (serovar Grippotyphosa, strain Andaman) was extracted and quantified by UV absorbance. All cultures were diluted to 40 pg/µL, and a ten-fold dilution series from 8 to 0.0008 pg/µL was made for each strain. Numbers of genomes were estimated based upon the size of the Pomona genome.

### Microscopic Agglutination Testing (MAT)

*Leptospira interrogans* serovars Autumnalis, Australis, Canicola, Copenhageni (Icterohaemorrhagiae), and Pomona, *Leptospira kirschneri* serovar Grippotyphosa, *Leptospira borgpeterseni* serovar Ballum, and *Leptospira* spp. of serogroup Hebdomadis, were procured from the National Veterinary Services Laboratories (NVSL, Ames, IA, USA) and maintained at 30°C in Polysorbate-80 bovine serum albumin medium (NVSL, Ames, IA, USA). MAT was performed as per the method of Cole et al [19]. Equal volumes of diluted canine sera were mixed with live leptospiral cultures, incubated at 30°C for 2 h, and examined under a dark-field microscope.

## Results

### Development and Laboratory Validation of the Canine Pathogenic *Leptospira* spp. FRET-PCR

We obtained all available *lig* gene sequences for canine pathogenic leptospires in GenBank for alignment and identified a target region that is highly conserved among these taxa (Fig. 1), but does not exist in saprophytic leptospires. By nucleotide megablast similarity search, each of the four oligonucleotides showed 18 hits with pathogenic leptospires, with 0–1 nucleotide mismatch, but no hits with other pathogens, non-pathogenic leptospires, or the host genome. The *Leptospira* species identified by nucleotide BLAST and gene accession numbers are shown in Table 1 [15], [20]–[24].

**Table 1:** Pathogenic canine leptospiral strains detectable by the *Leptospira* spp. *lig* FRET-PCR as indicated by sequence matches of primers and probes.

Species	Serogroup	Serovar	Strain	Gene accession #^a^
*L. interrogans*	Icterohaemorrhagiae	Copenhageni	Fiocruz L1-130	AE016823.1
*L. interrogans*	Icterohaemorrhagiae	Lai	56601	AE010300.2
*L. interrogans*	Icterohaemorrhagiae	Lai	IPAV	CP001221.1
*L. interrogans*	Pomona	Pomona	Kennewicki PO-06047	EU700267.1
*L. interrogans*	Pomona	Pomona	Kennewicki PO-06047	EU700268.1
*L. interrogans*	Pomona	Pomona	Kennewicki Cornell	AF534640.1
*L. interrogans*	Pomona	Pomona	Kennewicki	AF368236.1
*L. interrogans*	Pomona	Pomona	Kennewicki	U95056
*L. interrogans*	Pomona	Pomona	pLPLIGB	FJ030916.1
*L. interrogans*	Pomona	Pomona	pLPLIGA	FJ030617.1
*L. interrogans*	Canicola	Canicola	Kito	EU700267.1
*L. interrogans*	Canicola	Canicola	Kito	EU700268.1
*L. interrogans*	Canicola	Canicola	Hond Ultrecht IV	EU289225.1
*L. interrogans*	ND	ND	FPW1039	AKWR02000178.1
*L. interrogans*	ND	Manilae	UP-MMC-NIID	AB098516.1
*L. interrogans*	ND	Manilae	UP-MMC-NIID	AB098517.1
*L. kirschneri*	Grippotyphosa	Grippotyphosa	RM52	EF517920.1
*L. kirschneri*	Grippotyphosa	Grippotyphosa	Moskva V	AY190126.2

a Each of the four oligonucleotides in the *lig* FRET-PCR was used in nucleotide megablast searches (optimized for highly similar sequences) against all accessioned sequences in GenBank, and all matched to these 18 strains of pathogenic leptospires with 0–1 nucleotide mismatch.

**Figure 2:**
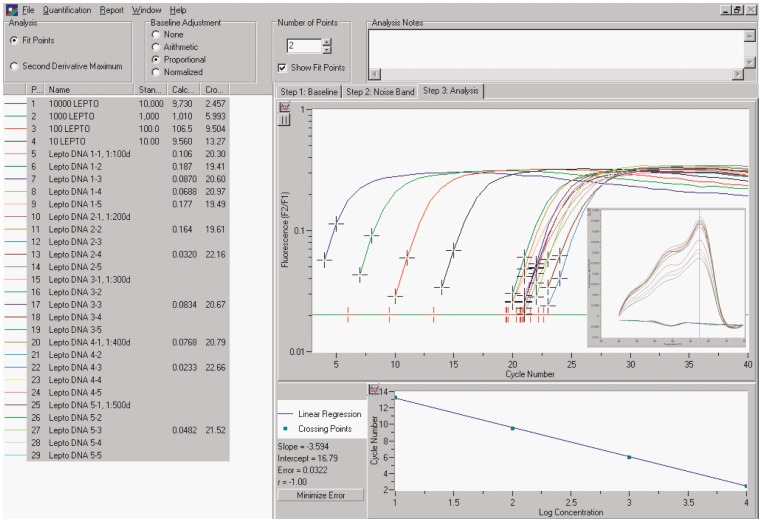
Detection limit of the *Leptospira* spp. *lig* FRET-PCR. For use as quantitative standard in the *lig* PCR, DNA from a pure culture of a wild-type isolate of *Leptospira interrogans* serovar Pomona was extracted and quantified by PicoGreen DNA fluorescence assay (Invitrogen, Karlsbad, CA, USA). The number of genomes was calculated assuming a *Leptospira* spp. genome size of 4.63 Mb (MW = 3.06×10^9^ Dalton), which was further used to determine the sensitivity of the *lig* FRET-PCR by amplification of logarithmic dilutions of this standard. To calculate the actual number of *lig* gene targets per leptospiral genome, the 10-copy standard DNA was further serially diluted from 1∶100-fold (0.1 genomes/PCR) to 1∶500-fold (0.02 genomes/PCR), and 5 aliquots of each dilution step were subjected to PCR detection. This approach yielded a Poisson distribution of positive and negative amplification reactions, with fewer of the 5 aliquots positive at higher dilutions. Logistic regression analysis indicated a 50% probability of a positive PCR at a PCR input of 0.0476 genomes. The complete separation between positive and negative reactions indicates a robust PCR methodology that reliably amplifies single target copies. Thus, we calculated from the mean of 0.5 targets per 0.0476 genomes an approximate number of 10.5 targets per leptospiral genome. The insert of the melting curves shows that positive amplification of leptospiral DNA is associated with the presence of a specific melting point (*T*_m_ = 62.5°C) which does not exist in the samples free of leptospiral DNA.

The *lig* FRET-PCR amplified genomic DNA extracted from *L. interrogans* Pomona with high sensitivity and specificity. *L. interrogans* Pomona DNA was diluted on a high background salmon sperm DNA, resulting in 0.2 µg salmon sperm DNA per 20 µL PCR. Negative controls of PCRs with background salmon sperm DNA never resulted in positive amplifications. Two-fold serial dilutions of leptospiral genomic DNA yielded either fully positive PCRs equivalent to PCRs with high target copy input, or negative amplification reactions (Fig. 2). At higher dilutions, not all of the 5 PCR reactions with equal dilution of the leptospiral DNA tested positive, indicating a Poisson distribution of the targets, with the positive PCRs containing 1 or more targets while the negative PCRs contained no copies of the target DNA (Fig. 2). This conclusion is justified based on the unambiguously positive or negative PCR amplification curves, indicating a robust PCR method that is capable of saturation amplifying single target molecules (Fig. 2). The resistance to PCR inhibition is indicated by the conditions of the PCR with very high background of unrelated DNA, resulting in amplification of single target molecules at a more than trillion-fold excess of unrelated DNA (2×10^−7^ g background DNA/1.93×10^−19^ g mass of 176 bp target DNA = 1.04×10^12^). The absence of any PCR inhibition is also evident in the equal slope of the amplification curve of low-copy samples as compared to high-copy samples (Fig. 2).

Logistic regression analysis of the amplification data in Figure 2 indicated a 50% probability of a positive PCR at a PCR input of 0.0476 genomes. Thus, we calculated from the mean of 0.5 targets per 0.0476 genomes an approximate number of 10.5 targets per leptospiral genome. The positive amplification of leptospiral DNA is associated with the presence of a specific melting curve (*T*_m_ = 62.5°C), which was the same for all 5 *Leptospira* strains tested, but does not exist in the samples free of leptospiral DNA (Fig. 2, insert), indicating 100% specificity. The tripartite melting curve results from different melting temperatures of the oligonucleotide species among the degenerate FRET probes.

### Comparison of Sensitivity between *lig* FRET, 16S rRNA TaqMan and lipL32 SYBR PCRs

Sensitivity of the lipL32 SYBR assay, 16S rRNA TaqMan assay and *lig* FRET PCR was compared using 10-fold serial dilutions of genomic DNAs from five different pathogenic leptospires. All were tested in triplicate, and no difference in sensitivity between strains was seen for the FRET-PCR or TaqMan PCR assays. The *lig* FRET-PCR showed 100% sensitivity when 0.008 pg DNA (approximately 1.6 genomes; equivalent to 3 *lig* gene copies per genome) was used per reaction and detected 7 of 15 (47%) replicates at the 0.0008 pg (0.16 genomes per reaction) concentration. The 16S rRNA TaqMan assay displayed sensitivity as published, detecting 9 of 15 (60%) replicates at the 1.6 genomes per reaction concentration but only 1 of 15 replicates (7%) at 0.16 genomes per reaction. No-template controls were consistently negative for both the FRET and TaqMan assays.

The lipL32 SYBR assay was only tested using *L*. interrogans serovar Pomona DNA. A specific product was produced from highly concentrated templates with a *T*_m_ of 83.5–84.0°C, but detection was erratic when using 16 or fewer genomes per reaction (Table 2). Primer-dimers (*T*_m_ 78–79°C) were seen at concentrations with <160 genomes per reaction. Several false positive results were noted, with melting temperatures suggestive of primer-dimers, including in template-free negative controls.

**Table 2:** Diagnosis of leptospirosis in survival canine cases.

	Case #1: April 2012	Case #2: April 2013	Case #3: May 2013
**Age and sex**	2 yr Neutered Male	7 mo Intact Male	1.5 yr Intact Male
**Type of Case**	Chronic	Chronic	Acute
**Blood PCR: FRET/Taqman**	−/−	−/−	+/−
**Urine PCR: FRET/Taqman**	+/+^a^	+/−	+/+^b^
**Post-Treatment PCR**	−/−	−/−	−/−
**Acute serology: MAT**	Not Done	Negative	Canicola 1∶100
**Convalescent serology: MAT**	Negative	Canicola 1∶800	Pomona 1∶400 Autumnalis 1∶200 Grippotyphosa 1∶100

a C_T_ for FRET assay 34.7. C_T_ for TaqMan assay 37.1.

b C_T_ for FRET assay 31.7. C_T_ for TaqMan assay 34.5.

### Diagnostic Validation of the *lig* FRET-PCR for Detection of Canine Leptospirosis

Once established and laboratory-validated as the best-performing PCR assay, the *lig* FRET-PCR was applied to diagnosis of pathogenic *Leptospira* spp. infections using 207 whole blood samples and 9 urine specimens from 207 dogs seen at the Ross University School of Veterinary Medicine Veterinary Teaching Hospital. All specimens were processed using standard DNA extraction procedures optimized and validated for diagnostic PCRs in the Auburn University Molecular Diagnostics Laboratory [16]. From these 207 dogs, 204 dogs were negative by the *lig* FRET-PCR. Three dogs contained leptospiral DNA in the urine, and one of these dogs with acute clinical disease was also positive in blood; the other two urine-positive dogs with chronic disease remained negative in blood (Table 2). The specimens of the three *lig* FRET-PCR-positive dogs were also analyzed using the 16S rRNA TaqMan PCR, the other laboratory-validated *Leptospira* spp. PCR with acceptable performance (Table 2), and the lipL32 SYBR PCR. Two of the urine specimens, but no blood samples, were positive by the 16S rRNA TaqMan PCR. In the lipL32 SYBR assay, all specimens of these three dogs remained negative.

The complete statistical analysis of the status and differential diagnosis of all dogs in this study, and of a larger sample of dogs in a follow-up study informed by the preliminary tests in this study will be reported in a separate communication. Here, we describe the full status of the 3 positive dogs because of the diagnostic interest of these rare leptospiral infections:

Case #1: On April 4, 2012, a two-year old, neutered mixed breed dog was seen at the RUSVM Teaching Hospital for proteinuria of one month duration. Prior testing had revealed an elevated urine protein:creatinine ratio of 2.63, consistent with glomerular-origin proteinuria. No remarkable findings were noted on physical examination. Complete blood count (CBC), serum biochemical panels and fecal floatation revealed no significant abnormalities. Whole blood was negative in real-time PCR tests for *Anaplasma*, *Babesia* and *Ehrlichia* species. Urine was clear, with a urine specific gravity (USG) of 1.029 and pH of 7.0. Proteinuria was detected using a urine dipstick (approximately 500 mg/dL) and confirmed using sulfosalicylic acid (SSA) precipitation. Electrophoresis showed the urine protein concentration was 389.9 mg/dL (normal range 10–50) with marked albuminuria and no gammopathy. Serum protein electrophoresis revealed high total protein (8.0 g/dL) with polyclonal gammopathy (1.66 g/dL, range 0.40–1.00) and a decreased albumin:globulin ratio, suggesting a chronic infectious agent. Blood and urine samples were collected for PCR testing using the LipL32 SYBR assay, 16S rRNA TaqMan assay, and the *lig* FRET-PCR assay. Using the *lig* FRET-PCR and the 16S rRNA TaqMan assays (Table 2), urine tested positive for pathogenic *Leptospira* spp. while blood tested negative, consistent with chronic infection. Both blood and urine samples were negative using the lipL32 SYBR assay.

Treatment with doxycycline (5 mg/kg q12hr for 21 days) was initiated on June 14, 2012, and a re-check examination was performed on July 6, 2012. At this time, blood and urine samples both tested negative by PCR, but proteinuria was still present. Serology, using MAT and representatives of eight Leptospira serogroups (Australis, Autumnalis, Ballum, Canicola, Copenhageni, Grippotyphosa, Hebdomadis, and Pomona), revealed no reactive antibodies. At a final re-check on November 1, 2012, the proteinuria had resolved (56 mg/dL).

Case #2: On April 16, 2013, a 7-month-old male Bull mastiff puppy was presented for acute onset of limping. It was afebrile and lameness was attributed to effusion in both stifles, possibly from osteochondrosis dissecans. During the course of testing, slightly elevated bilirubin (0.8 mg/dL, reference range 0.1–0.6) was noted, along with isosthenuria (USG 0.015), slight hematuria, and renal and squamous epithelial cells seen in urine sediment. CBC was unremarkable. Antibodies against *E. canis* or *Anaplasma phagocytophilum/platys* were not detected (4DX SNAP test; IDEXX, Westbrook, ME), and blood bacteriological cultures were negative. Urine and blood samples were subjected to pathogenic *Leptospira* spp. PCRs (Table 2); urine was positive by *lig* FRET-PCR, but not by 16S rRNA TaqMan or lipL32 SYBR PCRs, and blood tested negative in all assays. Following treatment with doxycycline, repeat PCR testing was negative for both blood and urine samples. The convalescent sample collected on May 14 was tested using MAT and did not contain antibodies to *Leptospira interrogans* serovar Canicola (Table 2).

Case #3: On May 13, 2013, a 1.5 year old, intact, male island mix dog presented for lethargy, inappetance, vomiting, and diarrhea of acute onset. The dog was parasitized by intestinal nematodes (*Trichuris* spp., *Ancylostoma* spp., and *Toxocara* spp.), and elevated eosinophils were noted on CBC (1×10^3^/µL), range 0.1–0.75). Mild monocytosis (1.5×10^3^/µL, range 0.15–1.35) was also seen. Biochemistry revealed several abnormalities, including hyperbilirubinemia (2.6 mg/dL), hypercalcemia (13.0 mg/dL, range 8.6–11.8) with hyperphosphatemia (16.5 mg/dL, range 2.9–6.6), and elevated creatinine (13 mg/dL, range 0.3–1.4). On urine analysis, hyposthenuria (USG 1.013) with proteinuria (500 mg/dL, confirmed using SSA precipitation) and hematuria were seen, with both red and white blood cells noted in urine sediment. A 4DX SNAP test was performed and no antibodies against *E. canis* or *Anaplasma phagocytophilum/platys* were detected. Serology was performed for *Leptospira*, using MAT; this acute serum reacted only with the *Leptospira interrogans* serovar Canicola strain (Table 2). Urine and blood tested positive in the *lig* FRET-PCR, only urine in the 16S rRNA TaqMan PCR, and blood and urine samples were negative in the lipL32 SYBR assay (Table 2). The dog was hospitalized for 6 days with intravenous fluids, after which oral doxycycline therapy was initiated. A recheck appointment was performed on May 28, at which time convalescent samples were PCR negative and the owner reported the dog was in good health. MAT results from convalescent serum collected May 28 revealed antibodies reactive to serovars Pomona, Autumnalis and Grippotyphosa (Table 2).

## Discussion

Canine leptospirosis is often difficult to diagnose, as it has a wide spectrum of clinical presentations, ranging from lethargy, depression, anorexia, and fever to diarrhea and icterus, which mimic the clinical findings of many other diseases including ehrlichiosis, babesiosis, canine distemper virus infection and canine brucellosis. Definitive diagnosis by culture and isolation of the organisms requires weeks, and seroconversion often does not occur until acute infection has resolved [25], [26]. Further, chronic seronegative carrier states have been reported [25], and the microscopic agglutination tests used for serology do not discriminate between infection and vaccination. PCR assays that enable the reliable detection of *Leptospira* DNA would be very useful for the detection of both acute and chronic infections. However, due to the minute amount of *Leptospira* DNA in the blood and urine specimens of infected dogs, a highly sensitive PCR platform is required for accurate diagnosis.

Choice of an appropriate PCR target gene and optimized design of primers and probes is absolutely critical to ensure the sensitivity and specificity of quantitative PCR [27]. Here, we developed a rapid and sensitive PCR assay targeting the *lig* genes, which exist only in pathogenic canine leptospires and not in saprophytic strains. Comparison of leptospiral genome numbers determined by DNA quantification and of *lig* gene copy numbers determined by *lig* FRET-PCR indicated variable numbers of *lig* gene copies per genome, with approximately 10 *lig* copies in our Pomona quantitative standard strain (Fig. 2), and approximately 3 *lig* copies in other pathogenic leptospiral field isolates (Table 2). The high numbers of 553 LigA protein and 914 LigB protein molecules per leptospiral cell [28] support a potential evolutionary benefit of *lig* gene duplication for pathogenic leptospirae.

The *lig* FRET-PCR assay was highly sensitive and enabled the specific detection of a single target gene copy, less than one genome of *Leptospira* spp. The multiple *lig* gene target copies per leptospiral genome may explain in part the high sensitivity of the *lig* FRET-PCR established in this study, aside from the robustness of the step-down amplification thermal protocol. Additionally, the *lig* FRET-PCR uses 10 µL of highly concentrated template DNA in each reaction, five times as much as has been validated for previous TaqMan or SYBR assays. Combining concentrated DNA extraction, increased template volume, and the sensitivity of the *lig* FRET-PCR, we could detect as few as 20 copies of the pathogenic leptospiral *lig* gene per one mL of blood or 10 copies per 3–4 mL of urine, translating into detection of single leptospiral bacteria per 0.17–0.5 mL of blood or 1 mL of urine. The whole process, from DNA extraction to completion and interpretation of PCR results, requires about 3 hours of time.

When we compared sensitivities using equal amounts of genomic DNA in each reaction, the *lig* FRET-PCR was almost 100-fold more sensitive than the widely accepted lipL32 SYBR assay and 10-fold more sensitive than a 16S rRNA TaqMan. The FRET-PCR successfully identified all five strains of *Leptospira* spp. tested, representing five different serogroups which cause canine infection. The specificity of the FRET and TaqMan PCR assays was 100% in all specimens tested. The lipL32 SYBR assay is unsuitable for amplification at low template concentrations, with primer-dimer formation resulting in reduced sensitivity and false-positive results. Altering the primer concentrations, reducing the annealing time, and using a different brand of master mix did not alleviate the problem (data not shown).

When applied to clinical samples from naturally infected dogs, the high sensitivity of the *lig* FRET-PCR allowed us to diagnose one chronic, seropositive case that was urine-negative in the 16S rRNA TaqMan PCR. FRET-PCR also confirmed the presence of *Leptospira* spp. in blood and urine of an acutely ill dog, whereas TaqMan was only able to detect the pathogen in urine, and both assays confirmed the presence of leptospires in the urine of an additional chronic, seronegative case. This underscores the importance of assay sensitivity in the diagnosis of both acute and chronic leptospirosis in dogs. Both the FRET and TaqMan assays confirmed that these dogs were negative for leptospiral DNA post-treatment. Thus, overall detection of canine leptospirosis was more sensitive with the *lig* FRET-PCR than with the 16S rRNA TaqMan PCR, which detected only 2 of the 3 cases, and the lipL32 SYBR PCR, which detected none of the 3 dogs with leptospirosis. Furthermore, analysis of urine specimens, despite greater difficulty obtaining these from frequently oliguric dogs with acute leptospirosis, is preferable over the analysis of the more easily obtainable blood specimens.

Our findings also underscore the limitations of serological testing for leptospirosis in dogs. Two of the three cases described here were confirmed, serologically, based upon convalescent serum; the third case was chronically infected but seronegative. Harkin et al [25] identified canine leptospirosis by PCR, but antibody against leptospires was not detected in the serum of the infected dog. Our findings confirm that serology is a poor predictor of urinary shedding of leptospires.

While we detected urinary shedding of leptospiral DNA in 3 dogs by the most sensitive *lig* FRET-PCR and are confident that these dogs represent true positive results, it is difficult to evaluate test accuracy by estimating the number of false negative PCR results in this study, or for that matter in any study, given the very high number of true negatives. One approach to estimate false negatives would be pattern matching in clinical appearance, biochemical parameters, and serology between dogs with confirmed leptospirosis and those that have remained negative by PCR. Another approach would be to maximize sample DNA input in the PCR by increasing the volume of urine for DNA extraction. This could be achieved by high-speed sedimentation of, e.g. 4 mL or more urine, and DNA extraction from the complete sediment. Such PCRs may have a DNA input equivalent to 1 mL or more urine as compared to the 0.1 mL equivalent used after standard direct extraction performed in this study. Such approaches will be required to not only evaluate PCR performance, but to estimate overall accuracy of diagnosis of canine leptospirosis, with the caveat that dogs with symptomatic leptospirosis may present with oliguria.

In conclusion, our study shows that the *lig* FRET-PCR established in this study is a reliable, rapid, and highly sensitive assay for the diagnosis of the most common pathogenic canine leptospires. To our knowledge, this is the first report of using FRET-PCR technology for the diagnosis of canine leptospirosis. The test shows better specificity than the widely used lipL32 SYBR, and higher sensitivity than the 16S rRNA TaqMan, and in particular the lipL32 SYBR assay, and can detect clinical cases of canine leptospirosis that could be overlooked using a less sensitive method. These data establish the *lig* FRET-PCR as best suited for PCR detection of canine leptospirosis, when used in combination with an optimized sample DNA extraction procedure. Further validation will require larger comparative studies with higher numbers of *Leptospira* spp.-positive animals that will allow firm establishment of negative as well as positive predictive values of this assay.
